# Exploring how health inequalities are conceptualised and measured in patient experience surveys in acute care: a protocol for a scoping review

**DOI:** 10.12688/hrbopenres.13998.1

**Published:** 2024-11-26

**Authors:** David Healy, John Gilmore, Jenny King, Jenny McSharry, Oonagh Meade, Éidín Ní Shé, Lorna Sweeney, Conor Foley, Chris Noone

**Affiliations:** 1 School of Psychology, University of Galway, University Road, Galway, H91 TK33, Ireland; 2 School of Nursing Midwifery and Health Systems, Health Sciences Centre, University College Dublin, Belfield, Dublin 4, D04 V1W8, Ireland; 3 Picker Institute Europe, Oxford, England, OX4 2JY, UK; 4 Graduate School of Healthcare Management, Royal College of Surgeons in Ireland, 123 St Stephen's Green, Dublin 2, D02 YN77, Ireland; 5 Health Information and Quality Authority, Cork, T12 Y2XT, Ireland

**Keywords:** health disparity, health inequity, patient-centred care, qualitative research

## Abstract

**Objective:**

The objective of this scoping review is to map measured and overlooked health inequalities in patient experience surveys in acute care and to explore the potential consequences of different conceptualisations of these health inequalities.

**Introduction:**

Measuring patient experience has become standard practice in many countries. However, despite the widespread awareness of the impact of health inequalities on various aspects of health, including patient experience, a comprehensive examination of whether and how health inequalities are measured in patient experience surveys has yet to be completed. The various ways in which these surveys conceptualise health inequalities may have important implications for how information about inequalities in patient experience is reported and used to allocate resources and plan quality improvement in health services. We will analyse the papers included in this scoping review to identify ways in which health inequalities have been conceptualised and measured in patient experience surveys in acute care and the potential consequences of framing health inequalities in different ways.

**Inclusion criteria:**

Papers that contain materials relating to patient experience measurement in any acute care context will be included. No limits will be placed on patient characteristics.

**Methods:**

A search strategy was developed with an information specialist. The database search will be limited to after September 2021. No limit will be placed on data sources. Grey literature searches will be completed and relevant experts will also be contacted to identify any patient experience surveys not captured through database or grey literature searches. Non-English papers will be included only if resources allow. Two independent reviewers will complete title and abstract, and full-text screening. Additional reviewers will resolve any conflicts. A data extraction form was developed. The extracted data will be analysed using Critical Discourse Analysis, a qualitative method used to examine how power, dominance and inequality are enacted in text.

## Introduction

### Rationale

Despite the growing research examining the impact of health inequalities on patient experience ( Bone *et al.*, 2014; Körner & Sizmur, 2013; Nuño-Solínis *et al.*, 2021), there is still no consensus on how best to conceptualize and measure these inequalities in patient experience surveys. Building on the work of Wolf and colleagues ( Wolf *et al.*, 2014; Wolf *et al.*, 2021), who have highlighted how patient experience continues to evolve as a concept, a recent concept analysis of patient experience identified 20 attributes which define patient experience ( Avlijas *et al.*, 2023). Examples of these attributes include respect for patients, comfort and pain, professionalism and trust, and hospital environment. The analysis drew on existing patient experience measurement tools and other literature that discussed patient experience more generally to define this concept. They define patient experience as, “…the combination of external and internal hospital processes, patient-centered attributes, patient-staff and staff-staff interactions during all episodes of care” ( Avlijas *et al.*, 2023, p. 20). Importantly, it differs from “patient satisfaction”, in that it is concerned with what happened to a patient during their time in a health facility and how it happened, rather than the patient’s personal expectations for the care provided to them ( Bull, 2021). Patient experience has evolved over the past two centuries, shaped by various innovations in medicine, patient advocacy and business management, eventually becoming a cornerstone of healthcare quality worldwide ( Adams *et al.*, 2024). Key evolutionary stages included the transition from the biomedical model of care, which placed priority on curing and managing disease, to a patient-centred model of care, which recognised the importance of the patient’s experience of care in the healthcare system. Partnerships formed between medical professionals and patients to incorporate the patient voice into the decision-making process, summarised succinctly by the widely used slogan in patient advocacy circles, “nothing about me, without me” ( Delbanco *et al.*, 2001).

System changes in global politics then began to influence patient care, notably through the adoption of the New Public Management principles during the 1980s ( Adams *et al.*, 2024). These principles advocated for greater dependence on market forces to run public services, requiring a greater need for evaluation processes to differentiate between the quality of services provided by different healthcare facilities ( Simonet, 2015). The experience economy emerged from these principles in the late 20 ^th^ century, proposing that businesses who provide a better healthcare experience could charge more for this service in a competitive market ( Pine & Gilmore, 1998). Despite the adoption of business management principles in healthcare throughout this period, there remained a focus on recognising the needs of the patients and how their experience can be improved through survey measures. Institutes such as Picker became experts in novel methodologies for measuring patient experience, developing the first patient experience survey for the National Health Service in the UK in the early 21 ^st^ century ( Jenkinson *et al.*, 2002). Since then, patient experience measurement has become standard practice in many countries across the world ( Adams *et al.*, 2024), seeking to further understand the various attributes of patient experience to support quality improvement initiatives in patient care.

However, there has been less work focusing on how patient experience varies across different communities. Terms such as health inequality, health inequity and health disparity are concepts commonly used interchangeably in the literature with respect to how health outcomes differ according to how people or communities are positioned in society. In their literature review and synthesis of previous definitions of health and health inequalities, McCartney and colleagues (2019) explored the varying definitions used to define each of these concepts in relation to health outcomes. They highlighted how different geographical regions use these terms differently, which can cause confusion. For example, in Europe, health inequality is used more commonly than health inequity to describe both terms. Arcaya and colleagues (2015) differentiate health inequality from health inequity in terms of social justice, where health inequity acknowledges the moral dimension of an inequality and the need to act on it – where inequality does not. They define health inequality as “any measurable aspect of health that varies across individuals or according to socially relevant groupings...” while health inequity or health disparity are defined as, “…a specific type of health inequality that denotes an unjust difference in health” ( Arcaya *et al.*, 2015, pp. 1–2). This “measurable aspect of health” can also apply to patient experience.

Examples of the effects of health inequalities and health inequities on patient experience has been explored across multiple contexts. A study examining the association between income disparities and patient-reported healthcare experience reported that, in a sample of 68,447 individuals in the USA who had a healthcare provider, lower income was repeatedly associated with poorer healthcare experiences compared to higher income ( Okunrintemi *et al.*, 2019). Results from a national survey in England reported that sexual minorities reported significantly worse patient experiences on four aspects of primary care (trust and confidence in doctor, doctor communication, nurse communication, and overall satisfaction) than heterosexual patients ( Elliott *et al.*, 2015). In a systematic review of 18 studies (n = 9,708 patients) examining racial, ethnic and socioeconomic differences in patient experience with clinician empathy, empathy scores were numerically lower for non-white patients compared to white patients ( Roberts *et al.*, 2021). Although none of these results were statistically significant, the authors suggest that the empathy gap for low socioeconomic and non-white individuals may be widening over time, with further research required to test such disparities. When examining richer, qualitative reports of health inequalities in patient experience, a participatory study exploring discrimination and abuse in public healthcare towards indigenous people living in rural Guatemala provided evidence of numerous ways in which minority groups can be mistreated in healthcare ( Cerón *et al.*, 2016). Examples included the denial of access to care due to the systems’ inability to communicate with them in their own language, being yelled at during consultations, and being lied to and poorly informed about the procedures being done to them. The authors conclude by emphasising a need to further explore the structural determinants of such discrimination and abuse towards indigenous peoples. Each of these studies recommend examining further how and why these disparities occur and dedicating efforts to improving the experiences of such groups in healthcare settings.

Health inequalities are commonly measured using equity stratifiers, referring to dimensions of equality which represent perceived inequality in healthcare provision ( Carroll *et al.*, 2021). They are generally categorical in form, such as demographic indicators associated with social, cultural and economic capital, to enable the measurement of structural inequalities by analysing aggregate data across different groups of people ( Carroll *et al.*, 2021). The PROGRESS-PLUS acronym (place of residence, race, occupation, gender, religion, education, socioeconomic status and social capital; O’Neill *et al.*, 2014) represents common sociodemographic indicators associated with health inequalities. The PLUS suffix describes additional characteristics related to age, disability, sexual orientation, relationship factors and time-dependent stratifiers that may describe other vulnerable and socially excluded groups. In contrast to research on inequalities in health outcomes, the measurement of indicators associated with inequalities in patient experience has been relatively limited to date. There has been only a number of, sometimes ambiguous, results available on stratifiers other than age ( Friedel *et al.*, 2023). In their review of health inequality measurement within health and social care data collections in Ireland, Carroll and colleagues (2021) also noted the limited use of many PROGRESS-PLUS equity stratifiers across different health contexts, with heterogeneous definitions frequently used. They call for agreed upon outcome measures of equity to allow for policy changes and interventions to be compared within and between populations.

Considering the lack of attention given to understanding health inequalities in patient experience measurement to date, this review aims to examine how health inequalities have been conceptualised and measured in patient experience surveys in an acute care setting and establish what the potential consequences of conceptualising and measuring health inequalities in different ways are. As the findings from patient experience surveys have important implications for quality improvement in patient care, mapping how these data are generated and discussing the implications of conceptualising patient experience survey items in different ways will help determine what more can be done to respond to health inequalities in patient experience. Acute care settings will be the focus of this review for two principal reasons. Firstly, the patient experience literature is large, with 153 patient experience measurement instruments identified across multiple contexts in a recent concept analysis of patient experience ( Avlijas *et al.*, 2023). As we wish to examine in detail how health inequalities are conceptualised and measured in patient experience surveys in a limited timeframe, focusing on a subset of contexts will enable a more thorough examination of the texts related to this process (e.g., guidance documents and technical reports of various surveys). Secondly, this review is part of a larger research project working with marginalised communities to make the National Inpatient Experience Survey in Ireland more accessible for, and increase participation in this survey among, marginalised groups. The National Inpatient Experience Survey collects data from patients who spend 24 hours or more receiving acute care in a public hospital in Ireland and are discharged during the survey period. The results of this review will directly inform later work completed on this project.

A scoping review methodology was chosen, as we aimed to identify and map existing evidence in the field and to clarify key concepts/definitions in the literature ( Munn *et al.*, 2018). A preliminary search of MEDLINE, the Cochrane Database of Systematic Reviews and JBI Evidence Synthesis was conducted and no current or underway systematic reviews or scoping reviews on the topic were identified.

### Objectives

This scoping review proposes two objectives to examine how health inequalities have been conceptualised and measured in patient experience surveys in acute care settings internationally:

- Map which health inequalities have been conceptualised and measured and which have been omitted or overlooked.
- Establish the potential consequences of conceptualising and measuring health inequalities in different ways.

### Review questions

In correspondence with the review objectives, the following review questions will be answered by the scoping review:

- Which health inequalities have been conceptualised and measured in the patient experience literature and which have been omitted or overlooked?
- What are the potential consequences of conceptualising and measuring health inequalities in different ways?

## Methods

The proposed scoping review will be conducted in accordance with the updated Joanna Briggs Institute (JBI) methodology for scoping reviews ( Peters *et al.*, 2020). The review protocol is reported in accordance with the Preferred Reporting Items for Systematic reviews and Meta-Analyses Protocols (PRISMA-P) Checklist ( Moher *et al.*, 2015; Shamseer *et al.*, 2015) with updates on best practice for reporting scoping review protocols also included (see the completed PRISMA-P checklist in the accompanying data repository available via the link in the "reporting guidelines" section below; Peters *et al.*, 2022). The review is pre-registered on the Open Science Framework repository using the generalised registration form for systematic reviews ( van den Akker *et al.*, 2023).

### Eligibility criteria

***Report characteristics.*** No limits will be placed on study design to ensure all relevant materials are identified. Only conference proceedings will be excluded as a publication source. Studies published in any language will be considered depending on resources available during the review. Authors of relevant non-English literature will be asked if it is feasible for them to translate their survey materials. Literature published after September 2021 will be included, with literature prior to this date accounted for through studies included as part of a previously published concept analysis of patient experience ( Avlijas *et al.*, 2023). This concept analysis completed a broad search of the patient experience literature up to September 2021, identifying materials we are interested in analysing as part of the current scoping review.

***Population.*** People who receive adult care in an acute health facility, or advocates for those people. By advocates we mean, a person who can offer advice, support or information to a health service user, or act on the behalf of the service user or their family, when dealing with a healthcare service. They can also represent the views of the service user when seeking information or making complaints where necessary ( HSE, 2024a). Literature reporting experience surveys conducted in a healthcare setting that do not measure patient experience, such as healthcare staff experience only or measures of patient satisfaction only, will be excluded.

***Concept.*** Materials that measure, or support the measurement of, patient experience will be examined in this review. Surveys and associated guidance and reporting documents will be examined. Attention will be given to identifying any adaptions made to preexisting surveys. Surveys routinely embedded in healthcare systems will be included. By routinely embedded, we mean surveys that are implemented and evaluated continuously over time (e.g., monthly, bi-annually, annually) as part of a healthcare system’s evaluation and quality improvement processes. Surveys not embedded in healthcare systems will be excluded. These may include once-off surveys developed as part of small-scale studies that are not implemented in practice over time. These eligibility criteria will be applied due to the scope of the larger project this review is part of, which focuses on updating a national survey embedded in the Irish healthcare system. Literature that does not detail sufficient information about the patient experience survey will be excluded. This will be determined on a case-by-case basis with justifications for exclusion provided. Efforts will be made to retrieve survey materials from authors where there is insufficient published data.

In terms of examining various dimensions of health inequalities, as discussed in the introduction, we consider it important to be able to distinguish between health inequality and health inequity when examining how different groups conceptualise these terms in their surveys (i.e., whether justice or fairness are considered in their measurement of different dimensions of inequality). As such, both terms will be used in this review to distinguish between conceptualisations that only measure variability in health across groupings (health inequality) and those that also consider whether these variabilities are unfair or unjust (health inequity or health disparity).

***Context.*** Any acute healthcare facility where patient experience is measured will be explored. By acute care we mean acute services that include all

“… promotive, preventive, curative, rehabilitative or palliative actions, whether oriented towards individuals or populations, whose primary purpose is to improve health and whose effectiveness largely depends on time-sensitive and, frequently, rapid intervention” ( Hirshon *et al.*, 2013 p. 386).

Acute care includes a wide range of functions within the healthcare system including, “…emergency medicine, trauma care, pre-hospital emergency care, acute care surgery, critical care, urgent care and short-term inpatient stabilization” ( Hirshon *et al.*, 2013 p. 386). Literature reporting experience surveys that are administered outside of a patient or acute care context will be excluded. If the volume of included studies becomes unfeasible to synthesise, the context will be narrowed to account for the time constraints and resources of the review team. Inpatient experience surveys will be prioritised in this instance due to the objectives of the wider project to which this scoping review pertains. Inpatient care refers to care, “…requiring overnight stay in hospital as well as care provided through day case services” ( HSE, 2024b). The wider project is discussed further in the “consultation with relevant stakeholders” section below.

### Information sources

Specific sources will include:

- Subject-specific online databases including MEDLINE via Ovid (Medical Literature Analysis and Retrieval System), CINAHL via EBSCOhost (Complete Cumulative Index to Nursing and Allied Health Literature), EMBASE via Elsevier and Psychological Information via EBSCOhost (PsycInfo).
- Forward and backward citation searches of included studies using the Citation Chaser tool ( Haddaway, 2021). This will include Avlijas and colleagues’ (2023) concept analysis of patient experience to capture relevant citations prior to the completion of this search.
- The “similar articles” function in PubMed will be used for included studies. This function uses its own algorithm that identifies articles that are similar to the original article.
- Expert stakeholder consultation. A contact list of international experts will be created by two authors (CF and LS) who work for the Health Information and Quality Authority (HIQA) – an independent body created to ensure high-quality and safe care for individuals utilizing health and social care services in Ireland. CF and LS will be able to provide a comprehensive list of international experts through their networks at HIQA. Members of the project steering committee with relevant expertise (e.g., researchers at Picker Europe and HIQA) will also help identify grey literature on specialist websites.

### Search strategy

Considerations were given to updating the search completed for the concept analysis of patient experience mentioned above ( Avlijas *et al.*, 2023). However, as our review is only interested in a subset of the literature identified through this concept analysis, it was decided that developing a more specific search tailored to the review topic would be a more efficient use of time and resources.

Published and unpublished literature will be identified through the search strategy. A three-step approach was used to develop the search strategy for this review. First, an initial limited search of CINAHL Complete and PsycInfo was undertaken to identify articles on the topic. These databases were used in a previous review with a similar topic to this review ( Beattie *et al.*, 2015). Second, the text words and index terms from titles and abstracts of relevant articles identified through this initial search were used to develop a full search strategy for this review. Search terms related to patient experience were combined with terms related to surveys. Finally, the full search strategy was developed for CINAHL (see Table 1). It will be adapted for each database where necessary. A more specific search was created as we are focused only on how health inequalities are conceptualised in patient experience surveys in acute care settings, rather than the concept of patient experience more generally. Search terms related to health inequality were not included in the search strategy to avoid overlooking relevant literature that does not explicitly refer to health inequality, or related terms, in-text. An information specialist at HIQA with expertise in search strategy development in a health context was consulted to support the selection of the databases and search terms. The Peer Review of Electronic Search Strategies (PRESS) Evidence-Based Checklist for peer review was used to assess the search strategy ( McGowan *et al.*, 2016; see the completed PRESS checklist in the accompanying data repository available via the link in data availability section below).

**Table 1.** Search strategy prepared for CINAHL in EBSCOhost.

#	Query
S11	S4 AND S7 AND S10
**Concept 3: acute care**	
S10	S8 OR S9
S9	TI (Hospital* OR "Ambulatory care" OR "Secondary care" OR "Acute care" OR inpatient*) OR AB (Hospital* OR "Ambulatory care" OR "Secondary care" OR "Acute care" OR inpatient*)
S8	(MH "Health Facilities+")
**Concept 2: Surveys or questionnaires**	
S7	S5 OR S6
S6	AB (patient* N3 (survey* OR questionnaire* OR measure* OR scale* OR assess* OR tool OR tools OR instrument*)) OR TI (patient* N3 (survey* OR questionnaire* OR measure* OR scale* OR assess* OR tool OR tools OR instrument*))
S5	(MH "Surveys+") OR (MH "Questionnaires+")
**Concept 1: Patient experience**	
S4	S1 OR S2 OR S3
S3	TI patient* N1 experience* OR AB patient* N1 experience*
S2	(MM "Patient Satisfaction")
S1	(MH "Patient Centered Care")

## Study records

### Data management

All citations identified will be imported into the Zotero reference management software ( Zotero, 2024).

### Study/Source of evidence selection

Citations will be imported to the proprietary screening software platform, Covidence ( Covidence systematic review software, n.d.), from Zotero for de-duplication and screening. An alternative open-source screening platform can be found in the “software availability” section below. The inclusion and exclusion criteria will be applied, and pilot tested with a random sample of papers by two reviewers, with the criteria clarified if needed. Any changes will be reported in the final review paper. Two reviewers will independently screen the titles and abstracts against the criteria. Disagreements will be discussed between the authors and, if necessary, with additional reviewers until consensus is achieved. Potentially relevant sources will be retrieved in full, and their citation details imported into Covidence. Full text screening will take place for these citations by two independent reviewers. Disagreements will be resolved through discussion or by additional reviewers. Reasons for inclusion or exclusion at the full-text screening stage will be reported in the scoping review and presented in a PRISMA flow diagram ( Page *et al.*, 2021).

### Data extraction

Data will be extracted from papers included in the scoping review by two independent reviewers using a data extraction tool developed by the reviewers in Covidence (see the data extraction protocol in the accompanying data repository available via the link in “data availability” section below). The tool was adapted from the data extraction template for scoping reviews published by JBI ( Peters *et al.*, 2020). The data extraction tool will be piloted by two independent reviewers and amended where necessary prior to commencing data extraction. Any changes will be reported in the final review paper. Any disagreements will be discussed or resolved by additional reviewers. If required, authors of included papers will be contacted to request missing or supplementary data.

Specific attention will be given to how health inequalities are conceptualised and measured in included literature. Equity stratifiers will be used to support the organisation of survey items relevant to health inequalities. Any survey items measuring PROGRESS-PLUS equity stratifiers will be extracted and synthesised to explore similarities and differences in groups included, and survey item wording used. Identified dimensions of inequality in patient experience that cannot be categorised under any of the PROGRESS-PLUS equity stratifiers (e.g., a question about the experience of discrimination in healthcare setting) will also be extracted and synthesised.

***Data items.*** The data items that will be extracted from each study include: population, context (e.g., study setting), author/s, date, title, source type (journal, book, website, etc.), volume, issue, pages, country of publication, aims/objectives, number of participants, study design, PROGRESS-PLUS equity stratifiers included in surveys or relevant survey materials (i.e., place of residence, race or ethnicity, occupation, gender or sex, religion, education, socioeconomic status, social capital, age, disability, sexual orientation, relationship factors, time-dependent stratifiers), data related to approaches to enhance the participation of minority or marginalised groups in patient experience surveys, other items included in survey, any rationale provided for conceptualisation and measurement of included survey items, and number of items in the survey.

### Data analysis and presentation

Extracted data will be presented in tables in the final report. Dimensions of inequality (e.g., PROGRESS-PLUS equity stratifiers) will be charted to highlight where these dimensions are included and excluded across included literature.

A critical discourse analysis (CDA) will then be conducted on the extracted data ( Fairclough, 2023; Mullet, 2018) to examine how “…social power abuse, dominance, and inequality are enacted, reproduced, and resisted by text and talk in the social and political context” ( Tannen *et al.*, 2015, p. 352). Mullet’s seven steps to CDA will be followed (see Table 2). As the first objective of this review is to examine how health inequalities are conceptualised and measured in patient experience surveys, CDA will enable a thorough analysis of how certain conceptualisations of the different dimensions of health inequalities may limit a survey’s, and by extension a patient’s, ability to adequately capture experiences of inequality in a healthcare setting. As CDA aims to provide an explanation for how and why such inequalities may persist, it further responds to this review’s second objective which aims to establish the potential consequences of conceptualising and measuring health inequalities in the various ways identified during the analysis.

**Table 2.** Mullet’s seven steps to critical discourse analysis ( Mullet, 2018).

Stage of analysis		Description	Example relating to current research
1	Select the discourse	Select a discourse related to injustice or inequality in society.	Discourses used in the development and reporting of patient experience surveys.
2	Locate and prepare data sources	Select data sources (texts) and prepare the data for analysis.	Systematic search of databases and grey literature identified through networks involved in managing patient experience surveys.
3	Explore the background of each text	Examine the social and historical context and producers of the texts.	Examine the social and historical context through which patient experience measurement has evolved and the producers of these texts.
4	Code texts and identify overarching themes	Identify the major themes and subthemes using choice of qualitative coding methods.	Draw upon interpretive repertoires relevant to health inequalities in patient experience and subject positions afforded to people who receive care in a health facility to be inductively coded following data familiarisation.
5	Analyse the external relations in the texts (interdiscursivity)	Examine social relations that control the production of the text; in addition, examine the reciprocal relations (how the texts affect social practices and structures). How do social practices inform the arguments in the text? How does the text in turn influence social practices?	Examine who is involved in the decision-making process regarding patient experience survey development (e.g., are patients reported to be involved at any stage) and what theory and empirical evidence informs the development of survey items (e.g., Marxian vs Weberian conceptions of social class).
6	Analyse the internal relations in the texts	Examine the language for indications of the aims of the texts (what the texts set out to accomplish), representations (e.g., representations of social context, events, and actors), and the speaker’s positionality.	Interrogate the structure or layout of the text, linguistic devices used to make a point (e.g., metaphor, rhetoric, etc.).
7	Interpret the data	Interpret the meanings of the major themes, external relations, and internal relations identified in stages 4, 5, and 6.	Generate themes that represent the interplay between the discourse analysed, dominant social practices and structures, and interpretative repertoires to explain how health inequalities are conceptualised and measured in patient experience surveys.

### Consultation with relevant stakeholders

JBI’s guidance for knowledge user engagement in scoping reviews will be followed for this review ( Pollock *et al.*, 2022). This review is part of the first work package of a research project which aims to facilitate collaboration between people from marginalised communities and relevant stakeholders within the Irish health service to co-design strategies for (1) enhancing the sensitivity of the National Inpatient Experience Survey in Ireland to health inequalities, and (2) increasing participation in the survey by members of marginalised communities. There are several reasons why this project is focused on health inequalities in relation to this survey, including the underrepresentation of marginalised groups such as the Traveller community in previous survey samples, the limited number of equity stratifiers included in the survey, and the survey's limited accessibility, with participants requiring a fixed address to receive the survey which is only available in digital or hardcopy formats. On this review to date, research partners at HIQA (CF and LS) have reached out to their international networks engaged in patient experience measurement in acute care to request materials relevant to this scoping review (i.e., patient experience surveys delivered in acute care and their supporting documentation). Once an initial analysis of the extracted data has been completed, the research team will meet to discuss preliminary findings from the review. The findings will then be discussed with patient and public involvement (PPI) representatives from the local community who are part of the project. The purpose of these meetings is to engage PPI representatives in the knowledge generation process, incorporating any insights they have into the analysis. Progress updates will also be provided to the project steering group who consist of researchers, representatives affiliated with the government health department and national health service in Ireland, and other community organisation representatives.

### Reflexivity

Reflexivity requires researchers to critically reflect on their role in generating knowledge throughout the research process, interrogating their own worldviews and what effects they might have on their interpretations of empirical data ( Braun & Clarke, 2013; Finlay, 2002). The lead author (DH) will keep a reflexive diary to record critical reflections during each step of the review process. Allowing space and time to engage in reflexivity in this way enables the researcher to continuously reflect on their research practice, particularly where there may not be others available to have these discussions at times ( Nadin & Cassell, 2006). The research team includes a wide range of experts from different fields including academic researchers in psychology and other health research disciplines and researchers working in patient experience programmes for independent research organisations. The potential impact of these roles on the review will be discussed in the final review write-up.

## Discussion

While there is recognition of the impact of health inequalities on various aspects of health, including their effects on patient experience ( Bone *et al.*, 2014; Körner & Sizmur, 2013; Nuño-Solínis *et al.*, 2021), there is fragmented evidence on how health inequalities are considered when designing patient experience surveys. Examining how health inequalities are conceptualised and measured in this context, and establishing what the potential consequences of these conceptualisations are, will provide stakeholders involved in designing, implementing and responding to patient experience surveys with further insights into how health inequalities may be currently overlooked in these surveys. It will also offer a basis for determining what can be done to make patient experience surveys more sensitive to health inequalities that may arise in patient settings.

## Study status

At the time of this protocol submission, the systematic scoping review search was being conducted in each of the chosen databases.

## Data Availability

No data are associated with this article. Open Science Framework Repository – Exploring how health inequalities are conceptualised and measured in patient experience surveys in acute care: a protocol for a scoping review. https://doi.org/10.17605/OSF.IO/FSEHK ( Healy & Noone, 2024) This OSF project contains the following extended data: PRESS Evidence-Based Checklist for peer review.docx Data_Extraction_Protocol.docx Extractor_instructions.docx Seven_steps_to_critical_discourse_analysis.docx Screener_instructions.docx Data are available under the terms of the Creative Commons Attribution 4.0 International Public License. Open Science Framework Repository – Exploring how health inequalities are conceptualised and measured in patient experience surveys in acute care: a protocol for a scoping review. https://doi.org/ 10.17605/OSF.IO/FSEHK ( Healy & Noone, 2024) This OSF project contains the following underlying data cited in text: PRISMA-P-checklist_with_scoping_review_updates.docx Data are available under the terms of the Creative Commons Attribution 4.0 International Public License.

## Software availability

The Rayyan open-source screening platform can be used to replicate the screening procedures described in this protocol.

Platform DOI: 10.1186/s13643-016-0384-4

## References

[ref-1] Adams C Harrison R Wolf J : The evolution of patient experience: from holistic care to human experience. *Patient Exp J.* 2024;11(1):4–13. 10.35680/2372-0247.1947

[ref-2] Arcaya MC Arcaya AL Subramanian SV : Inequalities in health: definitions, concepts, and theories. *Glob Health Action.* 2015;8(1): 27106. 10.3402/gha.v8.27106 26112142 PMC4481045

[ref-3] Avlijas T Squires J Lalonde M : A concept analysis of the patient experience. *Patient Exp J.* 2023;10(1):15–63. 10.35680/2372-0247.1439

[ref-4] Beattie M Murphy DJ Atherton I : Instruments to measure patient experience of healthcare quality in hospitals: a systematic review. *Syst Rev.* 2015;4(1): 97. 10.1186/s13643-015-0089-0 26202326 PMC4511995

[ref-5] Bone A Grath-Lone LM Day S : Inequalities in the care experiences of patients with cancer: analysis of data from the national cancer patient experience survey 2011–2012. *BMJ Open.* 2014;4(2): e004567. 10.1136/bmjopen-2013-004567 24531454 PMC3927926

[ref-6] Braun V Clarke V : Successful qualitative research: a practical guide for beginners. Sage,2013. Reference Source

[ref-7] Bull C : Patient satisfaction and patient experience are not interchangeable concepts. *Int J Qual Health Care.* 2021;33(1): mzab023. 10.1093/intqhc/mzab023 33533407

[ref-8] Carroll C Evans K Elmusharaf K : A review of the inclusion of equity stratifiers for the measurement of health inequalities within health and social care data collections in Ireland. *BMC Public Health.* 2021;21(1): 1705. 10.1186/s12889-021-11717-5 34538235 PMC8451151

[ref-9] Cerón A Ruano AL Sánchez S : Abuse and discrimination towards indigenous people in public health care facilities: experiences from rural Guatemala. *Int J Equity Health.* 2016;15(1): 77. 10.1186/s12939-016-0367-z 27177690 PMC4866428

[ref-10] Covidence systematic review software. Covidence. [Computer software]. (n.d.). Reference Source

[ref-11] Delbanco T Berwick DM Boufford JI : Healthcare in a land called peoplepower: nothing about me without me. *Health Expect.* 2001;4(3):144–150. 10.1046/j.1369-6513.2001.00145.x 11493320 PMC5060064

[ref-12] Elliott MN Kanouse DE Burkhart Q : Sexual minorities in england have poorer health and worse health care experiences: a national survey. *J Gen Intern Med.* 2015;30(1):9–16. 10.1007/s11606-014-2905-y 25190140 PMC4284269

[ref-13] Fairclough N : Critical discourse analysis. In: *The Routledge Handbook of Discourse Analysis.*2nd ed, Routledge,2023.

[ref-14] Finlay L : Negotiating the swamp: the opportunity and challenge of reflexivity in research practice. *Qual Res.* 2002;2(2):209–230. 10.1177/146879410200200205

[ref-15] Friedel AL Siegel S Kirstein CF : Measuring patient experience and patient satisfaction—how are we doing it and why does it matter? A comparison of European and U.S. American approaches. *Healthcare (Basel).* 2023;11(6):797. 10.3390/healthcare11060797 36981454 PMC10048416

[ref-16] Haddaway NR : citationchaser: an r package for forward and backward citations chasing in academic searching. (Version 0.0.1) [Computer software], *Zenodo*.2021. 10.5281/zenodo.4533747

[ref-17] Healy D Noone C : Exploring how health inequalities are conceptualised and measured in patient experience surveys in acute care: a protocol for a scoping review. [dataset].2024. 10.17605/OSF.IO/FSEHK PMC11895858 40070555

[ref-18] Hirshon JM Risko N Calvello EJ : Health systems and services: the role of acute care. *Bull World Health Organ.* 2013;91(5):386–388. 10.2471/BLT.12.112664 23678202 PMC3646345

[ref-19] HSE: Advocacy services. HSE.Ie.2024a. Reference Source

[ref-20] HSE: Inpatient/scheduled care. HSE.Ie.2024b. Reference Source

[ref-21] Jenkinson C Coulter A Bruster S : The picker patient experience questionnaire: development and validation using data from in-patient surveys in five countries. *Int J Qual Health Care.* 2002;14(5):353–358. 10.1093/intqhc/14.5.353 12389801

[ref-22] Körner K Sizmur S : Equal rights, equal respect: an examination of differential inpatient experience in the NHS. *Divers Equal Health Care.* 2013;10(4). Reference Source

[ref-23] McCartney G Popham F McMaster R : Defining health and health inequalities. *Public Health.* 2019;172:22–30. 10.1016/j.puhe.2019.03.023 31154234 PMC6558275

[ref-24] McGowan J Sampson M Salzwedel DM : PRESS Peer Review of Electronic Search Strategies: 2015 guideline statement. *J Clin Epidemiol.* 2016;75:40–46. 10.1016/j.jclinepi.2016.01.021 27005575

[ref-25] Moher D Shamseer L Clarke M : Preferred reporting items for systematic review and meta-analysis protocols (PRISMA-P) 2015 statement. *Syst Rev.* 2015;4(1): 1. 10.1186/2046-4053-4-1 25554246 PMC4320440

[ref-26] Mullet DR : A general critical discourse analysis framework for educational research. *J Adv Acad.* 2018;29(2):116–142. 10.1177/1932202X18758260

[ref-27] Munn Z Peters MDJ Stern C : Systematic review or scoping review? Guidance for authors when choosing between a systematic or scoping review approach. *BMC Med Res Methodol.* 2018;18(1): 143. 10.1186/s12874-018-0611-x 30453902 PMC6245623

[ref-28] Nadin S Cassell C : The use of a research diary as a tool for reflexive practice: some reflections from management research. *Qual Res Account Manag.* 2006;3(3):208–217. 10.1108/11766090610705407

[ref-29] Nuño-Solínis R Urtaran-Laresgoiti M Lázaro E : Inequalities in health care experience of patients with chronic conditions: results from a population-based study. *Healthcare (Basel).* 2021;9(8):1005. 10.3390/healthcare9081005 34442142 PMC8394123

[ref-30] Okunrintemi V Khera R Spatz ES : Association of income disparities with patient-reported healthcare experience. *J Gen Intern Med.* 2019;34(6):884–892. 10.1007/s11606-019-04848-4 30783877 PMC6544715

[ref-31] O’Neill J Tabish H Welch V : Applying an equity lens to interventions: using PROGRESS ensures consideration of socially stratifying factors to illuminate inequities in health. *J Clin Epidemiol.* 2014;67(1):56–64. 10.1016/j.jclinepi.2013.08.005 24189091

[ref-32] Page MJ McKenzie JE Bossuyt PM : The PRISMA 2020 statement: an updated guideline for reporting systematic reviews. *BMJ.* 2021;372: n71. 10.1136/bmj.n71 33782057 PMC8005924

[ref-33] Peters MDJ Godfrey C McInerney P : Best practice guidance and reporting items for the development of scoping review protocols. *JBI Evid Synth.* 2022;20(4):953–968. 10.11124/JBIES-21-00242 35102103

[ref-34] Peters MDJ Godfrey C McInerney P : Scoping reviews. In: E. Aromataris, C. Lockwood, K. Porritt, B. Pilla, & Z. Jordan (Eds.), *JBI manual for evidence synthesis*. JBI.2020;169:467–473. Reference Source

[ref-35] Pine BJ Gilmore JH : Welcome to the experience economy. *Harv Bus Rev.* 1998;76(4):97–105. 10181589

[ref-36] Pollock D Alexander L Munn Z : Moving from consultation to co-creation with knowledge users in scoping reviews: guidance from the JBI scoping review methodology group. *JBI Evid Synth.* 2022;20(4):969–979. 10.11124/JBIES-21-00416 35477565

[ref-37] Roberts BW Puri NK Trzeciak CJ : Socioeconomic, racial and ethnic differences in patient experience of clinician empathy: results of a systematic review and meta-analysis. *PLoS One.* 2021;16(3): e0247259. 10.1371/journal.pone.0247259 33657153 PMC7928470

[ref-38] Shamseer L Moher D Clarke M : Preferred Reporting Items for Systematic review and Meta-Analysis Protocols (PRISMA-P) 2015: elaboration and explanation. *BMJ.* 2015;349: g7647. 10.1136/bmj.g7647 25555855

[ref-39] Simonet D : The new public management theory in the british health care system: a critical review. *Adm Soc.* 2015;47(7):802–826. 10.1177/0095399713485001

[ref-40] Tannen D Hamilton HE Schiffrin D : The handbook of discourse analysis. John Wiley & Sons, Incorporated,2015. 10.1002/9781118584194

[ref-41] van den Akker OR Peters GJY Bakker CJ : Increasing the transparency of systematic reviews: presenting a generalized registration form. *Syst Rev.* 2023;12(1): 170. 10.1186/s13643-023-02281-7 37736736 PMC10514995

[ref-42] Wolf J Niederhauser V Marshburn D : Defining patient experience. *Patient Exp J.* 2014;1(1):7–19. Reference Source

[ref-43] Wolf J Niederhauser V Marshburn D : Reexamining “defining patient experience”: the human experience in healthcare. *Patient Exp J.* 2021;8(1):16–29. 10.35680/2372-0247.1594

[ref-44] Zotero: Zotero. (Version 6.0.37) [Computer software]. Corporation for Digital Scholarship.2024. Reference Source

