# Supplementary material for: Exploring how health inequalities are conceptualised and measured in patient experience surveys in acute care: a protocol for a scoping review
Source: HRB Open Res. 2026 Mar 9;7:74. Originally published 2024 Nov 26. [Version 3] doi: 10.12688/hrbopenres.13998.3 (PMC11895858; doi:10.12688/hrbopenres.13998.3)
Supplement: Peer review history

# Reviewer report: Reviewer response for version 1

**DOI:** 10.21956/hrbopenres.15364.r45924

**Published:** 2025-03-07

**Authors:** Mar Estupiñán Fdez. de Mesa

**Affiliations:** 1 University of Surrey, Surrey, UK

This study protocol aims to map out how inequalities in patient experience surveys is conceptualised and measured and the implications of these dimensions relating to examining differences among groups and informing quality improvement plans. The protocol is sounded and will cover a knowledge gap. However, the protocol will benefit from some revisions and further refinements. Below, I included some recommendations for authors' consideration.

 Recommendations:

 Research question 1: How will authors determine what has been omitted and overlooked in the papers included in the scoping review? These points imply a deliberate / accidental failure to include something or failure to notice or consider something on the part of the authors of those papers. The protocol authors' are recommended to reconsider research question 1.

 Since authors are interested in inequalities/inequities, they are recommended to check and include the PRISMA-EQUITY checklist for equity-focused reviews: https://www.prisma-statement.org/equity

 For clarity and ease of read, authors are recommended to display key eligibility criteria using bullet points/listing key criteria. They are also recommended to include a list of exclusion criteria.

 Regarding study design, authors are recommended to reconsider expanding the exclusion criteria. The aim of the research is to assess how inequalities are conceptualised and measured in patient experience surveys. Therefore, not only conference proceeding but also opinion articles, commentaries, editorials and their like do not seem relevant and may introduce subjective views from the respective authors. To this end, authors might want to reflect on limiting the inclusion criteria to patient satisfaction surveys, guidance and technical reports documenting how inequality-focused surveys were developed, methodological articles describing process to develop inequality indicators to measure patient experience using surveys. This is in line with the criteria authors included under 'concept:  Materials that measure, or support the measurement of, patient experience will be examined in this review.'

 Authors are recommended to reconsider the inclusion criteria regarding language. At present, it is unclear what process they will use to ensure a robust and transparent mechanism to translate relevant documents, how many authors will be involved to validated the process, etc. Authors might want to consider including only papers in the English language if this is the main language spoken by al research team members.

 Unless the authors' inclusion/exclusion criteria and search string is the same as that of Avlijas et al's paper, authors should consider expanding the search beyond 2021. Using different criteria and search string and searching publications from 2021 onwards means that authors may missed relevant documents.

 Table 1. Search strategy only shows terms relating to surveys or questionnaires. However, under characteristics and concept, authors suggested that they will include technical reports, guidance, and the like. How do authors plan to retrieve these documents in a way that is robust, systematic, and transparent?

 Reflexivity - since authors highlight the importance of equity and diversity, it would be useful to include a description of the team's demographic and not only the professional background (e.g., is the team ethnically and gender diverse?

Is the study design appropriate for the research question?

Yes

Is the rationale for, and objectives of, the study clearly described?

Yes

Are sufficient details of the methods provided to allow replication by others?

Yes

Are the datasets clearly presented in a useable and accessible format?

Yes

Reviewer Expertise:

Scoping reviews, mixed methods, patient care experience surveys, public health, policy, health systems and health services, equity/equality - inequity/inequality.

I confirm that I have read this submission and believe that I have an appropriate level of expertise to confirm that it is of an acceptable scientific standard, however I have significant reservations, as outlined above.

# Reviewer report: Reviewer response for version 1

**DOI:** 10.21956/hrbopenres.15364.r44776

**Published:** 2025-03-06

**Authors:** Margaret Greenfields

**Affiliations:** 1 Anglia Ruskin University, Cambridge, UK

I was very pleased to have the opportunity to review this extremely thorough and well-written submission outlining the protocol for a scoping review focused on how health inequalities are conceptualised and measured in patient experience surveys routinely administered to adult patients receiving care in acute settings. In preparing the protocol the authors have undertaken a preliminary search of MEDLINE, the Cochrane Database of Systematic Reviews and JBI Evidence Synthesis and have not identified any systematic or scoping reviews on the topic in question. In the designing for the scoping review the authors propose incorporating a very wide range of materials referring to patient experience surveys, excluding only conference proceedings and materials published after 2021. Somewhat ambitiously they also propose that materials not published in English should be included if feasible. Given the volume of materials which such an approach might generate mitigation steps to be taken should the volume of materials become unmanageable are incorporated into the design. The overall protocol as presented aligns to the updated Joanna Briggs Institute (JBI) methodology and reporting will utilise the PRISMA-P checklist. Importantly, materials will be interrogated to identify dimensions of inequality (identified through the use of  PROGRESS-PLUS equity stratifiers) and subjected to critical discourse analysis so as to highlight inclusions and omissions (as well as strengths and weakness in phrasing of questions) in patient satisfaction surveys. The authors propose that these methods will  enable in-depth analysis of how conceptualisations of health

 inequalities may limit the ability of the person completing the survey to adequately capture experiences of inequality in delivery and receipt of health care services.

**Detailed Comments Review**

**1. Is the rationale for, and objectives of, the study clearly described?**

 The authors have provided an  extremely detailed and plausible rationale for undertaking this scoping review, highlighting the varying ways in the terms ‘health inequality’ and ‘health inequity’ are utilised in different geographic regions, and reporting on a number of large-scale international studies which have identified how lower socio-economic status,  membership of particular ethnic or indigenous groups, or being a sexual minority are suggestive of worse patient experiences on a number of aspects of primary care, in particular  those associated with trust in clinicians and communication.  Additionally, abundant examples from smaller scale qualitative studies with marginalised or minoritised communities and individuals exist, which report on experiences of discrimination in healthcare when compared to majority or mainstream populations, and emphasise the necessity of undertaking additional research to understand how and why such disparities in patient experience occur.

 In identifying that measurement of inequalities in patient experience surveys in Ireland has been  limited, and that use of PROGRESS-PLUS equity stratifiers varies by context, the review team argue that mapping how such data gathering is conceptualised and generated offers considerable scope for  reducing health inequalities. The objectives of the study are very clearly defined and clearly aligned to their larger, overarching  project on  accessibility and  engagement with the National Inpatient Experience Survey in Ireland which seeks to gather more insights from marginalised or under-represented minority groups.

**2. Is the study design appropriate for the research question?**

 This is a highly ambitious (and important) scoping study focused on two research questions.

 a) Which health inequalities have been conceptualised and measured in the patient experience literature and which have been omitted or overlooked?

 b) What are the potential consequences of conceptualising and measuring health inequalities in different ways?

 Whilst Question A can clearly be met by utilising the research design as specified, Question B is likely to be significantly harder to answer. Whilst the design element of the study which includes wide-ranging stakeholder consultation, knowledge exchange with international partners and the development of an action plan to engage with marginalised populations is to be applauded, the question pertaining to the consequences of conceptualising and measuring health inequalities ‘differently’, feels as though it is very open-ended and potentially unanswerable as framed. Whilst such activities may enhance the quality and quantity of data gathered “consequences” as a concept reaches significantly further and may for example engage with  issues of training for clinicians with longitudinal impact which will require long-term monitoring. As such I would suggest that this protocol could perhaps be strengthened slightly by rephrasing the second research question to engage with a less wide-ranging term which focuses more on quality of data gathered and longer-term implications for capturing and improving patient experience.

 Overall this is an exceptionally well designed and innovative protocol, although I do have a few qualms about the potential breadth of the data to be included in the review, particularly pertaining to the feasibility of the inclusion on materials in languages other than English (or other languages spoken fluently/understand well by the research team). Although reference is made to the opportunity to invite researchers to offer translated materials I do feel that this is perhaps a ‘big ask’ of international colleagues and may not generate many responses.

 I would note too – that I am sure that if appropriate such materials will emerge within the review process - and there may be potential to explore data gathered by (for example) the European Fundamental Rights Agency who in their large-scale studies make repeated reference to health experiences of Roma, LGBT+, refugee and asylum seeking populations and frequently incorporate materials pertaining to the populations’ use of both in-patient (for example maternity care) and receipt of out-patient primary care services. Whilst such FRA studies may not fully align to the protocol parameters it might be worthwhile for the reviewers to explore these further and consider if such materials are appropriate to include within the review given the large-scale multi-country nature of FRA research, focused on populations of interest to the team. 

 Overall (with the caveat above re the feasibility of responding to  RQ2 as phrased and potential of incorporating international materials to be analysed) the design is robust and transparent.  I have a high level of confidence that as described the search strategy, data extraction and analysis will ensure reliability. Data management processes outlined in the protocol are appropriate.

**3. Are sufficient details of the methods provided to allow replication by others?**

 Yes largely, more detail could be provided with regard to the stakeholder consultation process and engagement and how this will support responding to Question 2, but more details are likely to emerge around the precise shape of these engagement and consultation activities as the project develops further.

**4. Are the datasets clearly presented in a useable and accessible format?**

 Not included although tables illustrate the search strategy for materials and the phased of critical discourse analysis to be undertaken. Data will be made available at the end of the project through the open source framework repository.

Is the study design appropriate for the research question?

Yes

Is the rationale for, and objectives of, the study clearly described?

Yes

Are sufficient details of the methods provided to allow replication by others?

Yes

Are the datasets clearly presented in a useable and accessible format?

Not applicable

Reviewer Expertise:

My area of expertise is predominantly in social/health policy pertaining to health inclusion of marginalised/minoritised populations. This includes a focus on best practice in community engagement and co-production to enhance health, My methodological specialism are in mixed-methods research and critical policy analysis, and I have experience in undertaking scoping reviews.

I confirm that I have read this submission and believe that I have an appropriate level of expertise to confirm that it is of an acceptable scientific standard.

# Reviewer report: Reviewer response for version 1

**DOI:** 10.21956/hrbopenres.15364.r44770

**Published:** 2025-02-24

**Authors:** Julie Broderick

**Affiliations:** 1 Trinity College Dublin, Dublin, Ireland

This protocol is well written and is an important area to interrogate. The authors are to be commended.

 Please address the following points to enhance the quality of this protocol.

**Abstract**

 End of introduction instead of ‘we will analyse’  - frame identify the purpose of the scoping review.

 Inclusion criteria –  could the word ‘material’ be reframed to refer to survey?

 Methods – identify which scientific databased will be searched  

 ‘’A data extraction form was developed’’ – rewrite in present tense.

**Introduction**

 Include more of a description about what patient surveys are in general– what do they normally ask about, how are they normally distributed and how often and at which points in the patient journey.

 Para beginning with ‘’Health inequalities are commonly measured…’’ could health stratifiers be explained including some examples.

**Objectives**

 Include an overall aim and rewrite objectives including ‘patient surveys’ – as reading them in isolation they are not clear in relation to this review. Also rewrite as ‘’ to explore …’’ etc

**Eligibility criteria**

 ‘’No limits will be placed on study design to ensure all relevant materials are identified’’ – I would advise to add in excluding reviews – as only primary studies should be included.

 It seems unrealistic to include studies from any language – if so specific searches would need to be conducted and resources allocated – so I would question the value and consistently in approach of the current plan. For this reason, I would advise English language only.

 Specific detail on how the grey literature will be searched is needed.

 Clarify who will conduct the search.

 Query amalgamating search from (Avlijas et al., 2023) - to me this seems unusual. The premise of the current survey would have to exactly map that of Avlijasso so search terms/engines exactly matched. Under the section ‘’Search Strategy’’ it is acknowledged that the current review focusses on a  subset of the review by Avlijasso. Therefore search terms would be different and engines listed would not appear to be matched. I suggest the authors run the full search or limit years included in review and bring in Aviljas review in the discussion section.

 Population – define age limit of adult

 General point – these types of surveys embedded in practice are unlikely to be the subject of publications, therefore how will these be sourced?

 In the section ‘’Consultation with relevant Stakeholders’’ = ‘’There are several reasons why this project is focused on health inequalities in relation to this survey, including the underrepresentation of marginalised groups such as the Traveller community in previous survey samples, the limited number of equity stratifiers included in the survey, and the survey’s limited accessibility, with participants requiring a fixed address to receive the survey which is only available in digital or hardcopy formats. ‘’ Could references be included to back up some of these statements where possible?

 ‘’The findings will then be discussed with patient and public involvement (PPI) representatives from the local community who are part of the project’’ – clarify if members of the PPI group will have lived experience of health inequalities.

Is the study design appropriate for the research question?

Yes

Is the rationale for, and objectives of, the study clearly described?

Yes

Are sufficient details of the methods provided to allow replication by others?

Yes

Are the datasets clearly presented in a useable and accessible format?

Yes

Reviewer Expertise:

NA

I confirm that I have read this submission and believe that I have an appropriate level of expertise to confirm that it is of an acceptable scientific standard, however I have significant reservations, as outlined above.
